# Preoperative serum bilirubin is an independent prognostic factor for curatively resected esophageal squamous cell carcinoma

**DOI:** 10.1186/s12885-023-11215-4

**Published:** 2023-07-28

**Authors:** Xiancong Huang, Yang Chen, Huan Yang, Ruting Wang, Zhongjian Chen

**Affiliations:** 1 Department of Thoracic Surgery, Zhejiang Cancer Hospital, Hangzhou, 310022 Zhejiang China; 2 Zhejiang Key Laboratory of Diagnosis & Treatment Technology on Thoracic Oncology (Lung and Esophagus), Hangzhou, 310022 Zhejiang China; 3 The Second Clinical Medical College of Zhejiang Chinese Medical University, Hangzhou, 310053 China; 4 Experimental Research Center, Zhejiang Cancer Hospital, Hangzhou, 310022 Zhejiang China

**Keywords:** Bilirubin, Unconjugated bilirubin, Prognosis, Anticancer

## Abstract

**Purpose:**

This study examines prognostic value of preoperative serum bilirubin, including unconjugated bilirubin (UCB), conjugated bilirubin (CB), and total bilirubin (TB), in esophageal squamous cell carcinoma (ESCC) patients who underwent curative resection.

**Methods:**

Between May 2010 and December 2012, a total of 351 ESCC patients were retrospectively reviewed. All the patients underwent curative resection as their primary treatment. Clinicopathological features and overall survival (OS) rate were investigated. Kaplan-Meier curves were used to calculate the OS rate, and the prognostic factors were identified by Cox regression model. Besides, the potential inhibition effect of UCB on ESCC was investigated with both in vitro and in vivo models.

**Results:**

The higher-level groups of UCB, CB, and TB demonstrated longer OS than their low counterparts, with hazard ratio (HR) values of 0.567 (95% CI: 0.424–0.759), 0.698 (95% CI: 0.522–0.933), and 0.602 (95% CI: 0.449–0.807), respectively. All three forms of bilirubin were identified as independent prognostic factors for patients with ESCC, and they were found to effectively stratify the survival risk of patients at TNM stage III. In vivo and in vitro models further confirmed the inhibitory effect of unconjugated bilirubin (UCB) on the proliferation of ESCC.

**Conclusion:**

The findings of our study have shed new light on the prognostic value and biological functions of bilirubin in relation to ESCC. These results may contribute to a better understanding of the underlying mechanisms involved in ESCC tumorigenesis and provide potential therapeutic pathways for treating ESCC.

**Supplementary Information:**

The online version contains supplementary material available at 10.1186/s12885-023-11215-4.

## Introduction

Esophageal squamous cell carcinoma (ESCC) accounts for about 90% of the 572,000 new esophageal cancer cases each year [1–4], and is the predominant subtype of esophageal cancer in China [4, 5]. Currently, surgical resection with lymphadenectomy is the major treatment for ESCC. Despite the breakthroughs in surgical technique and multidisciplinary strategy, ESCC has a poor prognosis, with a 5-year survival rate ranging from  10% to 25% [6]. At present, TNM staging system is mainly used to predict the prognosis of ESCC. However, the factors of TNM staging system, such as lymph node metastasis and invasion depth, are not available until surgery. Therefore, a more convenient, non-invasive biomarker is always desired to predict the treatment outcome and monitor ESCC progression.

Unconjugated bilirubin (UCB), an end product of heme catabolism, can be taken up into the liver and metabolized into conjugated bilirubin (CB) by the enzyme uridine diphosphoglucuronate glucuronosyltransferase 1A1 (UGT1A1) [7, 8]. In human, UCB and CB comprise 80% and 20% of total bilirubin (TB), respectively. Recently growing evidence reveals that bilirubin has many health benefits, such as anti-inflammation [9], anti-oxidation [10], and anticancer [11] properties. Several studies indicated an inverse correlation between serum bilirubin concentration and tumor incidence, including lung cancer [11–13], and colorectal cancer [14, 15], and lightly elevated serum bilirubin level is associated with better prognosis. UCB is the active form of bilirubin. In vitro experiments proved that UCB possessed anticancer effect on colon cancer [14] and human adenocarcinoma cells [16].

Bilirubin has been demonstrated as a promising biomarker, and has a potential anticancer effect in some cancers [14]. However, it is still unclear whether bilirubin has a similar role in ESCC. We hypothesized that curatively resected ESCC patients with a mildly elevated serum bilirubin level would have a longer survival. The three forms of bilirubin (UCB, TB, and CB) are all routinely determined in clinical laboratory test, and can be repeatedly collected during the whole cancer treatment process, making them potentially convenient biomarkers for ESCC prognosis, especially in the long-term monitoring. The present study aims to explore the prognostic significance of bilirubin in ESCC patients with curative resection, and to further evaluate its potential anticancer property in ESCC.

## Materials and methods

### Patients and clinical data collection

We retrospectively reviewed medical records of ESCC patients underwent radical resection at Zhejiang Cancer Hospital, from May 2010 to December 2012. The study protocol was approved by the Research Ethics Committee of Zhejiang Cancer Hospital (China), in which experiments were conducted in accordance with the ethical standards of the Helsinki Declaration of 1975, and written informed consent was obtained from all individuals. The inclusion criteria were: (1) pathologically diagnosed as ESCC, (2) surgery included radical resection, (3) no treatment before surgery, (4) preoperative laboratory testing available. Cases with the following criteria were excluded: (1) incomplete medical records, (2) died from non-cancer-related causes, (3) severe hepatobiliary (Child-Pugh score > 6) disease or hemolytic disease, and (4) with other malignancies. According to these criteria, there were 351 matched cases.

Information including age, sex, drinking habit, smoking habit, tumor grade, neural invasion, tumor thrombus, and pathological stage (TNM stage determined according to American Joint Committee on Cancer 8th edition) were collected. Preoperative biochemistry and blood testing was collected in fasting condition. Participants were followed up until December 2017, and overall survival (OS) was calculated from the date of surgery to the date of death or that of the last follow-up.

### Survival analysis of serum UCB, CB, and TB in ESCC patients

Receiver operating characteristic (ROC) curve analysis was conducted to assess the predictive ability of serum UCB, CB, and TB for determining the 5-year survival status. The optimal cutoff values were obtained by calculating the maximum Youden’s index values, which is the sum of sensitivity and specificity minus one. The participants were categorized into two groups, namely low-level and high-level groups, based on the optimal cutoff values of serum UCB, CB, and TB. The Kaplan-Meier curves were plotted, and the differences between low-level and high-level groups were compared using the log-rank test. To validate the findings, the median values and tertiles of serum UCB, CB, and TB were also utilized as cutoff values in the survival analysis. Furthermore, univariate Cox regression analysis was performed to evaluate the hazard ratio (HR) of UCB, CB, TB, and clinicopathological features. Meanwhile, multivariate Cox regression analysis was conducted to determine whether these bilirubin measurements were independent predictors of survival after adjusting for clinicopathological features. The normality of the variables was assessed using the Shapiro-Wilk test. The Wilcoxon test or Kruskal-Wallis test was used to compare bilirubin levels with different clinicopathological features. The correlation between bilirubin and other variables was determined using Spearman’s rank correlation. Statistical analysis was conducted using R (4.1.0), and plotting was carried out using Prism (9.2.0).

### Cell viability

Human ESCC KYSE150 and KYSE30 cell lines were purchased from Nanjing Kebai Biotechnology Co., Ltd. (Nanjing, China) in 2016, and authenticated by a short tandem repeat (STR) report by Shanghai biowing biotechnology Co., Ltd (Nanjing, China) in 2019. Cells were cultured in RPMI Medium 1640, which was supplemented with 10% fetal bovine serum (FBS), 100 units/mL of penicillin, and 100 µg/mL of streptomycin. The cells were maintained at 37 °C in a humidified incubator with 5% CO_2_. The cells were seeded at a density of 5 × 10^3^ cells/well in 96-well plates. After incubation for 24 h, different concentrations of UCB (0, 2.5, 5, 10, 20, 40 µM) were added to the cells, which were then incubated for 24 or 48 h. The MTT assay was performed according to the manufacturer’s instructions (Beyotime Biotechnology, Haimen, China) to evaluate cell viability. In addition, real-time cell proliferation analysis was performed by xCELLigence (RTCA) DP instrument (Roche Diagnostics GmbH, Mannheim, Germany). Cells were seeded in 16 well microplates (E-plates; Roche Diagnostics, Switzerland), at a density of 5 × 10^3^ cells/well for both KYSE150 and KYSE30 cells. UCB concentrations of 0, 5, 10, and 20 µM were added 24 h subsequent to seeding. The impedance was recorded at 15 min intervals for 120 h, and cell proliferation curves were drawn. UCB was obtained from Aladdin Reagent Co., Ltd. (Shanghai, China).

### Xenograft mouse model

Forty 5-week-old female BALB/c nude mice (16-19 g) were obtained from Shanghai SLAC laboratory Animal Co., Ltd. (Shanghai, China). Animal experiments were conducted according to the National Institutes of Health Guide for Care and Use of Laboratory Animals. A total of 3 × 10^6^ KYSE30 cells were suspended in 200 µL of RPMI Medium 1640 containing 10% Corning® Matrigel® (NY, USA) and injected into the flank of the mice. When the average tumor volume reached over 50 mm^3^, the xenograft mouse models were randomly and evenly divided into four groups: a vehicle group (20% 2-Hydroxypropyl-β-cyclodextrin) and three UCB treatment (17.5, 35, and 70 mg/kg) groups. The high dose of 70 mg/kg UCB was chosen according to the paper, in which 75 mg/kg was a non-toxic dose for mouse [17]. Intraperitoneal administration of UCB and measurement of tumor size (length and width) and body weight were conducted every 2 days. After 16 days of UCB intervention, mice were euthanized using pentobarbital sodium, followed by cervical dislocation. At the end of the experiment, blood was collected through the orbital venous sinus, and the sera were separated and stored at -80 °C. The tumors were harvested, weighed, and fixed, embedded in paraffin, and then sliced into tissue Sect. (4 µM). After hematoxylin-eosin (HE) staining, a pathologist reviewed all the slides for tumor differentiation and mitotic cells.

## Results

### Patient characteristics

A total of 351 patients were enrolled in this study, their demographics and characteristics were listed in Table 1. There are 293 (83.5%) males, 245 (69.8%) smokers, and 229 (65.2%) drinkers. The distribution of pathological TNM stages(pTNM) was: I, 68 (19.4%); II, 102 (29.1%); III, 151 (43.0%); IV, 30 (8.5%). The distributions of serum UCB, CB, and TB concentrations were listed in Fig. S1, and none of them matched a normal distribution. Median values of serum UCB, CB, and TB were 7.9, 4.8, and 12.9 µM, respectively (Table 1**)**.

**Table 1:** Demographics and characteristics of patients in the study

Parameters^a^		Population (%) n = 351
Age (year)	Mean/median (range)	61/61 (43–81)
Sex	Male	293 (83.5)
Smoking	Yes	245 (69.8)
Drinking	Yes	229 (65.2)
Tumor thrombus	Yes	91 (25.9)
Neural invasion	Yes	125 (36)
Tumor grade	G1(well differentiation)	35 (10.0)
	G2(moderately differentiation)	291 (82.9)
	G3(poorly differentiation)	25 (7.1)
pN stage^a^	N0	160 (45.6)
	N1	114 (32.5)
	N2	47 (13.4)
	N3	30 (8.5)
pT stage^a^	T1	52 (14.8)
	T2	66 (18.8)
	T3	222 (63.2)
	T4	11 (3.1)
pTNM stage^a^	TNM I	68 (19.4)
	TNM II	102 (29.1)
	TNM III	151 (43.0)
	TNM IV	30 (8.5)
UCB (µM)^b^	Mean/median (range)	8.36/7.9 (1.0-27.6)
CB (µM)^b^	Mean/median (range)	5.24/4.8 (1.2–16.2)
TB (µM)^b^	Mean/median (range)	13.6/12.9 (3.4–43.8)

^a^: pN stage, pT stage and pTNM stage were determined according to American Joint Committee on Cancer 8th edition. ^b^: UCB: unconjugated bilirubin; CB: conjugated bilirubin; TB: total bilirubin. The normal ranges of UCB, CB, and TB are 0–13, 0–7, and 0–20 µM, respectively

### Prognostic significance of serum UCB, CB, and TB in ESCC patients

**Fig. 1:**
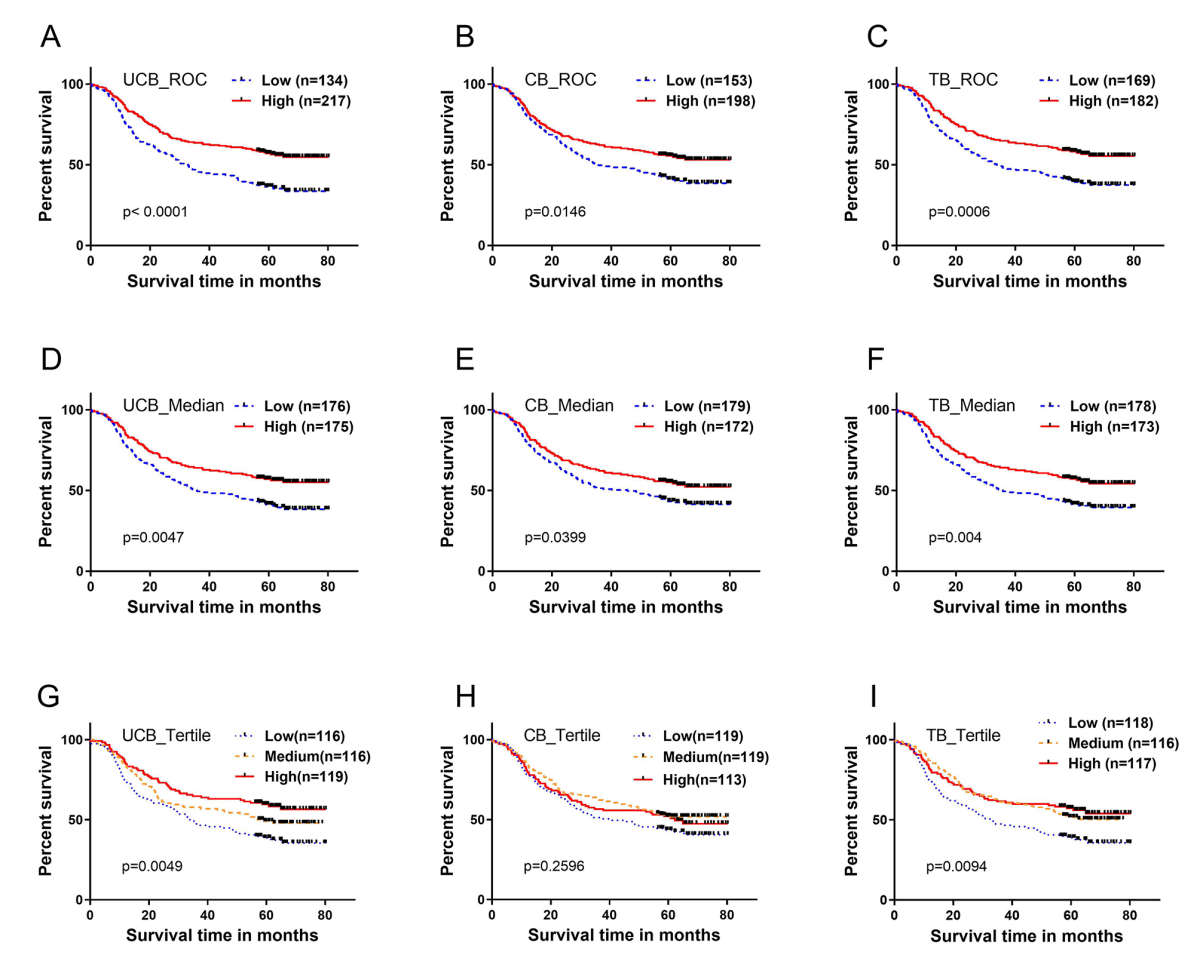
Survival analysis: Kaplan-Meier curve (log rank test) showed that serum unconjugated bilirubin (UCB), conjugated bilirubin (CB), and total bilirubin (TB), were associated with the overall survival (OS) in ESCC patients. **(A-C):** when population was divided into low (≤ cutoff) and high (> cutoff) level groups with optimal cutoff values of UCB (6.75 µM), CB (4.55 µM), and TB (12.6 µM), from ROC curve analysis, respectively; **(D-F):** when population was split into low and high level groups with median values of UCB (7.9 µM), CB (4.8 µM), and TB (12.9 µM), respectively; **(G-I):** when population was split into tertiles

ROC curve analysis was used to determine the optimal cutoff values for three serum markers: UCB, CB, and TB, which were found to be 6.75, 4.55, and 12.6 µM, respectively (as shown in Fig. S2). Based on these cutoff values, the study population was divided into low- and high-level groups. The high-level group for each of these markers was found to have a significantly better outcome compared to their low-level counterparts, as demonstrated in Fig. 1 (panels A-C). Specifically, the high-level group had a higher 5-year survival rate of 44.2% compared to 26.1% in the UCB group, 42.4% compared to 30.7% in the CB group, and 44.0% compared to 31.2% in the TB group, respectively. The prognostic significance of the serum markers was assessed based on their p-values, with UCB found to have the strongest prognostic significance, followed by TB and CB. Further survival analysis using a median-split approach showed that UCB, CB, and TB remained significant prognostic factors (Fig. 1: **D-F**). In addition, when the study population was divided into tertiles based on the concentration of the three forms of bilirubin, a slight UCB concentration-dependent trend in survival was observed, while no similar trend was observed between the concentration of CB (or TB) and survival (Fig. 1: **G-I**).

### Univariate and multivariate Cox regression analysis

In the univariate Cox analysis, several factors were identified as significant prognostic factors, including tumor grade, neural invasion, tumor thrombus, pT stage, pN stage, pTNM stage, UCB, CB, and TB, as shown in Table 2. Among them, bilirubin showed a protective role in survival, with hazard ratio (HR) values of 0.567 (95% confidence interval [CI]: 0.424–0.759) for UCB, 0.698 (95% CI: 0.522–0.933) for CB, and 0.602 (95% CI: 0.449–0.807) for TB. Furthermore, multivariate Cox regression analysis including UCB and major clinicopathological features such as tumor grade, neural invasion, tumor thrombus, and pTNM stage, showed that UCB was an independent biomarker from pTNM stage and other features. Similarly, CB and TB were also separately identified as independent biomarkers from pTNM stages and other clinicopathological features, as shown in Table S1.

**Table 2:** Univariate and multivariate Cox regression analysis ^a^

**Features**	Univariate Cox		Multivariate Cox	
	HR(95%CI)	p Value	HR(95%CI)	p Value
Sex	0.971(0.660–1.429)	0.880	-	-
Age	1.113(0.832–1.489)	0.472	-	-
Smoking habit	0.943(0.689–1.289)	0.711	-	-
Drinking habit	1.188(0.871–1.620)	0.277	-	-
Tumor grade	1.829(1.278–2.620)	0.00097	1.285(0.840–1.965)	0.248
Neural invasion	1.931(1.442–2.586)	< 0.0001	1.315(0.970–1.781)	0.077
Tumor thrombus	1.794(1.315–2.448)	0.0002	1.296(0.938–1.791)	0.116
pT stage	1.527(1.247–1.870)	< 0.0001	-	-
pN stage	1.862(1.612–2.151)	< 0.0001	-	-
pTNM	2.057(1.703–2.484)	< 0.0001	1.809(1.469–2.227)	< 0.0001
UCB	0.567(0.424–0.759)	0.0001	0.605(0.451–0.812)	0.0008
CB	0.698(0.522–0.933)	0.0151	-	-
TB	0.602(0.449–0.807)	0.0007	-	-

^a^: Factors included in the multivariate model were UCB and pTNM stage, tumor grade, neural invasion, and tumor thrombus, which had significant prognostic values in the univariate Cox analysis; UCB: unconjugated bilirubin; CB: conjugated bilirubin; TB: total bilirubin

### Combining bilirubin with pTNM stage improved the survival prediction accuracy and further stratified the risk in ESCC patients

**Fig. 2:**
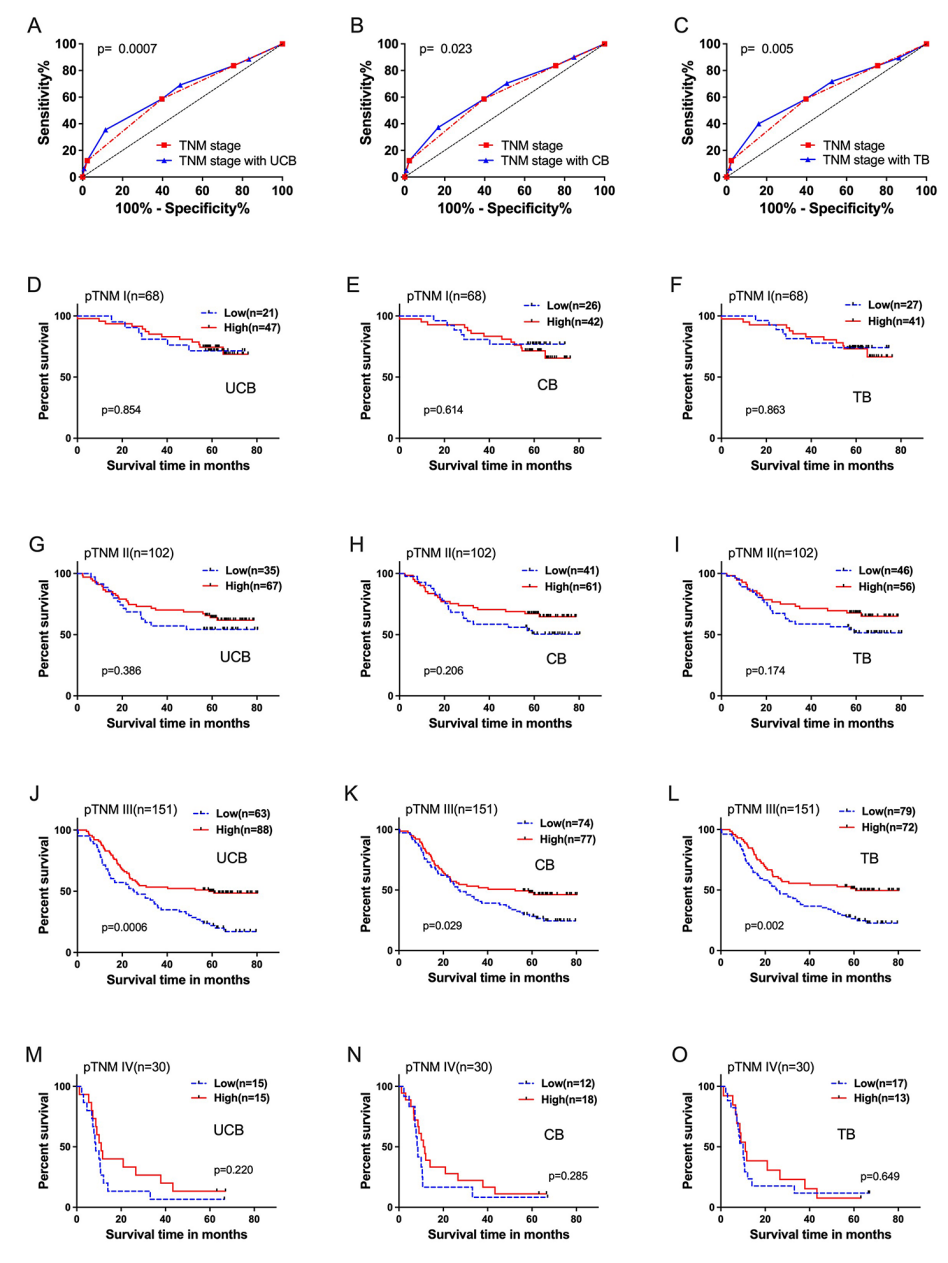
ROC curves used to evaluate the predictive accuracy of 5-year survival rates. **(A)** pTNM stage alone and combined with serum UCB; **(B)** pTNM stage alone and combined with serum CB; **(C)** pTNM stage alone and combined with serum TB. Further stratification in risk by UCB **(D, G, J, M)**, UC **(E, H, K, N)**, and TB **(F, I, L, O)** levels for different pTNM stages

A multivariate Cox regression model was used to analyze UCB and pTNM stage, with regression coefficients of -0.516 and 0.710, respectively. A patient’s risk score was then derived using the following formula: risk score = (-0.516 × level of UCB + 0.710 × level of pTNM stage) [18]. ROC curves were drawn for pTNM stage and the above risk score (combination of UCB and pTNM), and then their area under the curve (AUC) of ROC curves were compared using the method established by DeLong et al., [19]. When combined with UCB, the prediction accuracy of pTNM stage was significantly improved from to 0.614 (95%CI: 0.554–0.673) to 0.643 (95%CI: 0.586–0.701) (Fig. 2: **A**). In subgroups analysis of different pTNM stages, UCB had significant stratification for pTNM III patients (Fig. 2: **D, G, J, and M**). Likewise, the prediction performance of pTNM stage was significantly improved when combined with CB or TB, with ROC values increased from 0.634 (95%CI: 0.576–0.692) to 0.639 (95%CI: 0.581–0.697) and 0.643 (95%CI: 0.585-0.700), respectively (Fig. 2: **B, C**). Similarly, both CB and TB were able to further stratify the risk of pTNM III patients (Fig. 2: **E, H, K**, and **N for CB, F, I, L** and **O for TB**).

### Correlation between serum bilirubin biochemical parameters and clinicopathologic features

**Fig. 3:**
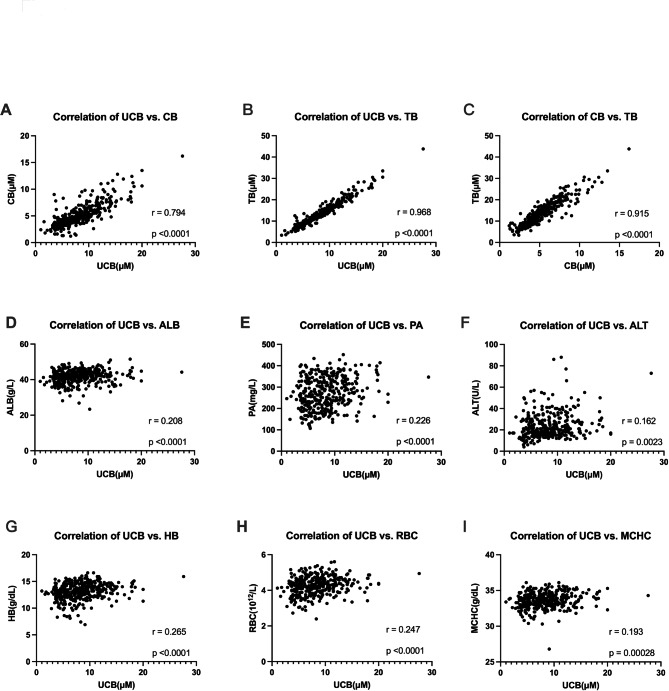
Correlation plotting between the three forms of bilirubin **(A-C)** and correlation between serum UCB and other biochemical parameters **(D-I)**.

There were significantly correlations among UCB, CB, and TB (Table 3; Fig. 3A-C). We analyzed the correlation between bilirubin and blood testing parameters related to liver function, kidney function and blood cell status. The result showed that the concentration of UCB, CB, and TB had a significantly positive correlation with that of albumin (ALB), alanine aminotransferase (ALT), mean corpuscular hemoglobin concentration (MCHC), hematocrit (HCT), red blood cell count (RBC), hemoglobin (HB) **(**Table 3**and** Fig. 3D-I**).** Regarding clinicopathologic features, patients with serum UCB levels above 6.75 µM had a lower percentage of lymph node metastasis compared to those with lower UCB levels (50.2% vs. 61.2%, p = 0.0478, Fig. S3), while CB and TB showed a non-significant trend. However, UCB, CB, and TB were not found to be significantly associated with other clinicopathologic features in this study.

**Table 3:** Correlation analysis of serum UCB, CB, and TB with biochemical parameters and blood testing parameters in ESCC patients^a^

Parameters		UCB	CB	TB
UCB	r	1.000	0.794	0.968
	p value	NA	< 0.0001****	< 0.0001****
CB	r	0.794	1.000	0.915
	p value	< 0.0001****	NA	< 0.0001****
TB	r	0.968	0.915	1.000
	p value	< 0.0001****	< 0.0001****	NA
ALB	r	0.208	0.135	0.192
	p value	< 0.0001****	0.0114*	0.0003***
PA	r	0.226	0.103	0.187
	p value	< 0.0001****	0.0557	0.0004****
AST	r	0.082	0.066	0.084
	p value	0.126	0.221	0.115
ALT	r	0.162	0.133	0.161
	p value	0.0023**	0.0127*	0.0024**
TBA	r	-0.014	-0.030	-0.013
	p value	0.795	0.575	0.805
MCV	r	-0.052	-0.029	-0.044
	p value	0.331	0.587	0.41
MCHC	r	0.193	0.178	0.197
	p value	0.00028***	0.00082***	< 0.0001****
MCH	r	0.047	0.062	0.057
	p value	0.381	0.246	0.29
RDW	r	-0.076	-0.092	-0.081
	p value	0.158	0.086	0.13
HCT	r	0.205	0.174	0.204
	p value	0.00011***	0.0010**	0.00012***
RBC	r	0.247	0.173	0.232
	p value	< 0.0001****	0.0011**	< 0.0001****
HB	r	0.265	0.200	0.256
	p value	< 0.0001****	0.00017**	< 0.0001****

^**a**^: Spearman correlation was used, and *: ***p*** < 0.05, **: ***p*** < 0.01, ***: ***p*** < 0.001, ****: ***p*** < 0.0001; UCB: unconjugated bilirubin; CB: conjugated bilirubin; TB: total bilirubin; ALB: albumin; PA: prealbumin; AST: aspartate aminotransferase; ALT: alanine aminotransferase; TBA: total bile acid; MCV: mean corpuscular volume; MCH: mean corpuscular hemoglobin; MCHC: mean corpuscular hemoglobin concentration; RDW: red blood cell distribution width; HCT: hematocrit; RBC: red blood cell count: HB: hemoglobin; r: correlation coefficient

### UCB inhibited the viability of ESCC cells

**Fig. 4:**
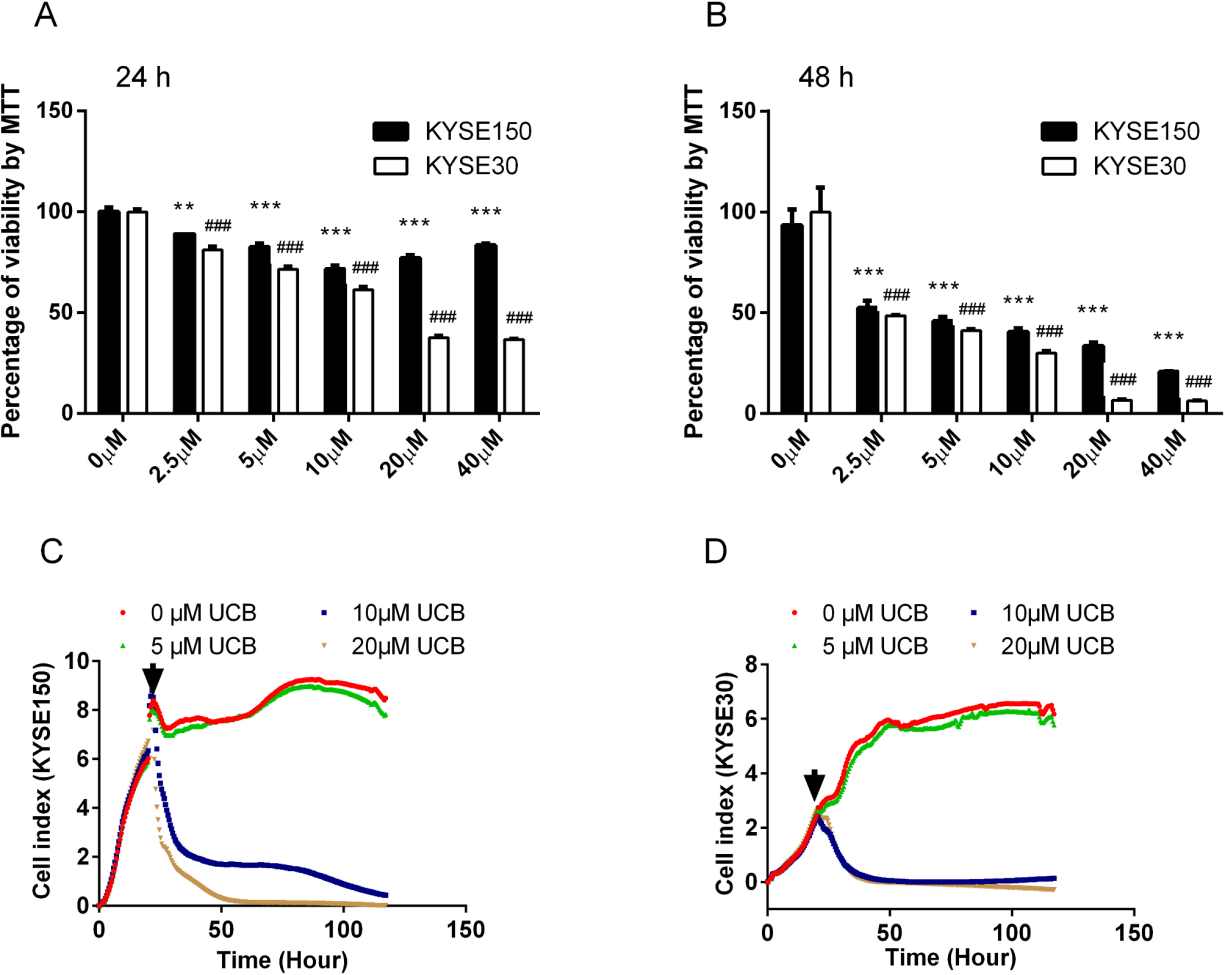
UCB inhibited the proliferation of ESCC cell lines. Cell viability of KYSE150 and KYSE30 cells was assessed using MTT assay after treatment with 0, 2.5, 5, 10, 20, and 40 µM UCB for 24 h **(A)** and 48 h **(B).** The data are presented as mean + SEM. The cell index-time curves were obtained for KYSE150 **(C)** and KYSE30 **(D)** when treated with 0, 5, 10, and 20 µM UCB for 120 h using the xCelligence RTCA system. The arrows indicate the time of adding bilirubin, and the data are presented as mean. Two-tailed Student’s t-tests were performed between the 0 µM UCB group and other level groups in KYSE150 and KYSE30 cells. The results are denoted as ** for p < 0.01, *** for p < 0.001, and ^###^ for p < 0.001. SEM stands for standard error of the mean

The MTT assay results demonstrated that UCB concentrations of 2.5, 5, 10, 20, and 40 µΜ significantly inhibited the viability of ESCC KYSE150 and KYSE30 cells in a dose-dependent manner when incubated for 24 and 48 h, as shown in Fig. 4A and B. Physiological concentrations of UCB (2.5, 5, and 10 µΜ) showed inhibition rates of 47.5%, 54.1%, and 59.5%, respectively, on KYSE150 cells and inhibition rates of 51.5%, 58.9%, and 69.9%, respectively, on KYSE30 cells when treated for 48 h incubation. Furthermore, the cell index values obtained from xCelligence RTCA system indicated that 10 and 20 µM UCB had a significant anticancer effect on both ESCC cell lines (Fig. 4C-D).

### UCB inhibited esophageal tumor growth in xenograft mouse model

**Fig. 5:**
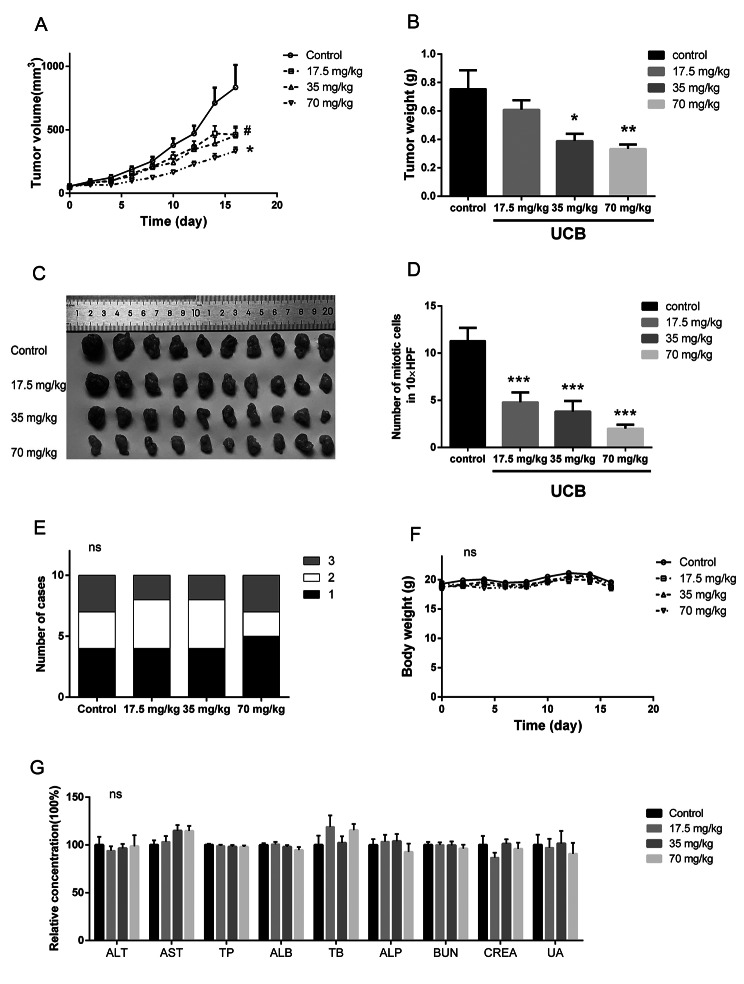
UBC delayed the tumor growth in xenograft mouse model. **(A)** Tumor volume-time curves, **(B)** tumor weight, **(C)** image of collected tumors, and **(D)** number of mitotic cells in 10 high power fields, showed a significant inhibition effect of UCB on tumor growth in vivo. **(E)** There was no change in tumor grade levels after treating UBC in vivo. **(F)** Body weight-time curves showed no significant systemic toxicity, and **(G)** serum chemistry test, indicated no significant liver toxicity or kidney toxicity after administration of UCB to mice. Liver function related parameters: alanine transaminase (ALT), aspartate aminotransferase (AST), total protein (TP), albumin (ALB), total bilirubin (TB), alkaline phosphatase (ALP); kidney function related parameters: blood urea nitrogen (BUN), creatinine (CREA), uric acid (UA). Data are expressed as mean + SEM. A two-tailed Student’s t-test was used to compare tumor volume, tumor weight, body weight, and chemistry values between each treated group and the control group. A two-tailed Mann Whitney test was used to compare the number of mitotic cells between each treated group and the control group. A Chi-Squared test was used to compare grade levels among the groups. ng: p > 0.05, *:p < 0.05, **:p < 0.01, ***: p < 0.001. SEM: standard error of the mean

The tumor volume-time curve showed that 35 and 70 mg/kg of UCB significantly inhibited the tumor growth, with a reduction of 45.5% and 60.0% compared to the control in the last measurement, respectively, while 17.5 mg/kg UCB had no significant inhibition (Fig. 5: **A, C**). Similarly, the average tumor weights of 35 and 70 mg/kg groups were significantly decreased by 48.5% and 66.0%, respectively (Fig. 5: **B**). The pathological analysis indicated UCB treatment significantly reduced the number of mitotic cells (Fig. 5: **D**), while did not influence the tumor grade levels (Fig. 5: **E**). No significant toxicity was observed in body weight-time curves or blood chemistry testing in this study (Fig. 5: **F, G)**.

## Discussion

Though increasing evidence reported mildly elevated concentrations of bilirubin are associated with cancer initiation and progression, few studies have directly proven the relationship between the prognostic significance and its anticancer property [12, 14–16]. Our study not only investigated the prognostic value of bilirubin in patients with curatively resected ESCC, but also further evaluated its potential anticancer role using in vitro and in vivo models. We found that: (1) higher preoperative serum bilirubin levels (including UCB, CB, and TB) were associated with longer survival in ESCC patients; (2) patients with UCB levels greater than 6.75 µM had a lower percentage of lymph node metastasis; (3) physiological concentrations of UCB had an anticancer effect in ESCC.

Due to the limitation of the TNM staging system in predicting the prognosis of ESCC, high-throughput methods, such as genomics [20], proteomics [21], and metabolomics [22] are wildly used to discovery promising biomarkers for ESCC. However, these methods are time-consuming and costly. In contrast to the existing methods, our study evaluated serum bilirubin as a convenient and low-cost approach in predicting OS of curatively resected ESCC patients. In the past, different forms of bilirubin, either UCB, or CB, or TB, have been reported as prognostic biomarker for cancers [11, 15]. Our study showed that the three forms of bilirubin had positive effect on survival, with UCB as the most significant one. Higher serum UCB concentrations were significantly associated with the decreased risk of lymph node metastasis, and our data implied a slight UCB concentration-dependent survival in ESCC. It might be explained by that UCB is the main antioxidant and anticancer form of bilirubin [10, 14].

Though UCB is reported to be an anticancer metabolite, it is unclear whether UCB within physiological range (0–13 µM) could have anticancer effect in ESCC cells. McGeary and colleagues discovered UCB as an antioxidant at concentrations of 16–17 µM [23], and UCB concentrations of 25 [14] and 17 µM [16] were reported to have anticancer effect in cancer colon cells and human adenocarcinoma, respectively. Our study demonstrated that UCB concentrations of 2.5, 5, and 10 µM, significantly inhibited the viability of ESCC cells in a concentration-dependent manner, indicating that ESCC cancer cells are sensitive to UCB. Meanwhile, xenograft mouse model showed that UCB could significantly delay the growth of tumor in a dose-dependent manner. Together, our in vitro or in vivo data demonstrated UCB had anticancer effect in ESCC, which could, at least partially, explain the reason why patients with higher bilirubin levels, especially UCB levels, had better prognosis in ESCC patients. There is a concern regarding the mechanism underlying the anticancer effect of bilirubin. There are several studies reported that the anticancer effects of bilirubin are associated with increased reactive oxygen species caused by bilirubin [16], and that bilirubin induces apoptosis of colon adenocarcinoma cells by directly dissipating mitochondrial membrane potential [14]. However, the detailed molecular mechanisms, especially in ESCC, are needed to be investigated in the further.

Since bilirubin is proved to be a healthy metabolite in vivo, there is a potential issue about the factors influencing bilirubin homeostasis. It is well-know that liver is responsible for bilirubin metabolism and elimination, and thus hepatobiliary diseases, including parenchymal liver disease, alcoholic liver disease, and fatty liver disease, can affect bilirubin levels in the body. The cases in this study were surgical patients who underwent liver function assessment prior to surgery, which excluded patients with severe liver problems. Therefore, in future studies evaluating the clinical significance of serum bilirubin, it is important to take into consideration liver function assessment. In addition to hepatobiliary disease, there are two factors that have been associated with bilirubin concentrations in humans. On one hand, UCB is a metabolite of heme, which is mainly derived from hemoglobin found in red blood cells. Therefore, the status of red blood cells is closely associated with UCB levels [7]. Our data showed that serum bilirubin level was positively associated with red blood cell related parameters, such as red blood cell count (RBC), hemoglobin (HB), hematocrit (HCT), and mean corpuscular hemoglobin concentration (MCHC). On the other hand, UCB is poorly soluble in water at physiologic pH, and mainly bound to albumin (ALB) in circulation [24], our data further showed that ALB and pre-albumin (PA) concentrations are slightly correlated with bilirubin levels in vivo (Table 3). It is worthy to explore the regulation mechanisms for bilirubin homeostasis in depth of in the future.

There were limitations in our present study. On one hand, our study population is relatively of small size, focusing curatively resected ESCC patients, and from one center. A large-scale population-based multi-center study, which includes more cases in TNM I, II, and IV, is needed to confirm the prognostic significance of bilirubin in ESCC. On the other hand, we did not demonstrate detailed molecular mechanisms for the anticancer effect of UCB in ESCC in the present study.

Our study identified preoperative serum UCB, CB, and TB as useful and convenient prognostic biomarkers in patients with curatively resected ESCC, and also discovered the anticancer effect of UCB in this disease. These findings provide a better understanding of the biological processes underlying ESCC and offer a novel insight into the potential biological function of bilirubin in this condition.

## Electronic supplementary material

Below is the link to the electronic supplementary material.

Supplementary Material 1

Supplementary Material 2

Supplementary Material 3

Supplementary Material 4

## Data Availability

Available from the corresponding author on reasonable request.
