# Supplementary material for: Preoperative serum bilirubin is an independent prognostic factor for curatively resected esophageal squamous cell carcinoma
Source: BMC Cancer. 2023 Jul 28;23:706. doi: 10.1186/s12885-023-11215-4 (PMC10375695; doi:10.1186/s12885-023-11215-4)
Supplement: Supplementary file 1 — Supplementary Material 1 [file 12885_2023_11215_MOESM1_ESM.docx]

**Table S1**: **Multivariate cox regression analysis result for CB and TB^a^**

| **Features** | **Multivariate cox^b^** | |  | **Multivariate cox^c^** | | |
| --- | --- | --- | --- | --- | --- | --- |
|  | **HR** | ***p* Value** |  | **HR** | ***p* Value** |  |
| Tumor grade | 1.394(0.912-2.131) | 0.126 |  | 1.344(0.878-2.059) | 0.174 |  |
| Neural invasion | 1.292(0.954-1.749) | 0.098 |  | 1.309(0.967- 1.773) | 0.0819 |  |
| Tumor thrombus | 1.288(0.932-1.779) | 0.125 |  | 1.279(0.925-1.768) | 0.137 |  |
| pTNM stage | 1.827(1.484-2.248) | <0.0001 |  | 1.800(1.462-2.216) | <0.0001 |  |
| CB | 0.721(0.539- 0.965) | 0.0276 |  | - | - |  |
| TB | - | - |  | 0.652(0.486-0.875) | 0.00433 |  |

^a^: CB: conjugated bilirubin; TB: conjugated bilirubin; ^b^: Factors included in the multivariate cox analysis were CB and clinicopathological features; ^c^: Factors included in the multivariate cox analysis were TB and clinicopathological features.
