# Supplementary figures and images for: Preoperative serum bilirubin is an independent prognostic factor for curatively resected esophageal squamous cell carcinoma
Source: BMC Cancer. 2023 Jul 28;23:706. doi: 10.1186/s12885-023-11215-4 (PMC10375695; doi:10.1186/s12885-023-11215-4)

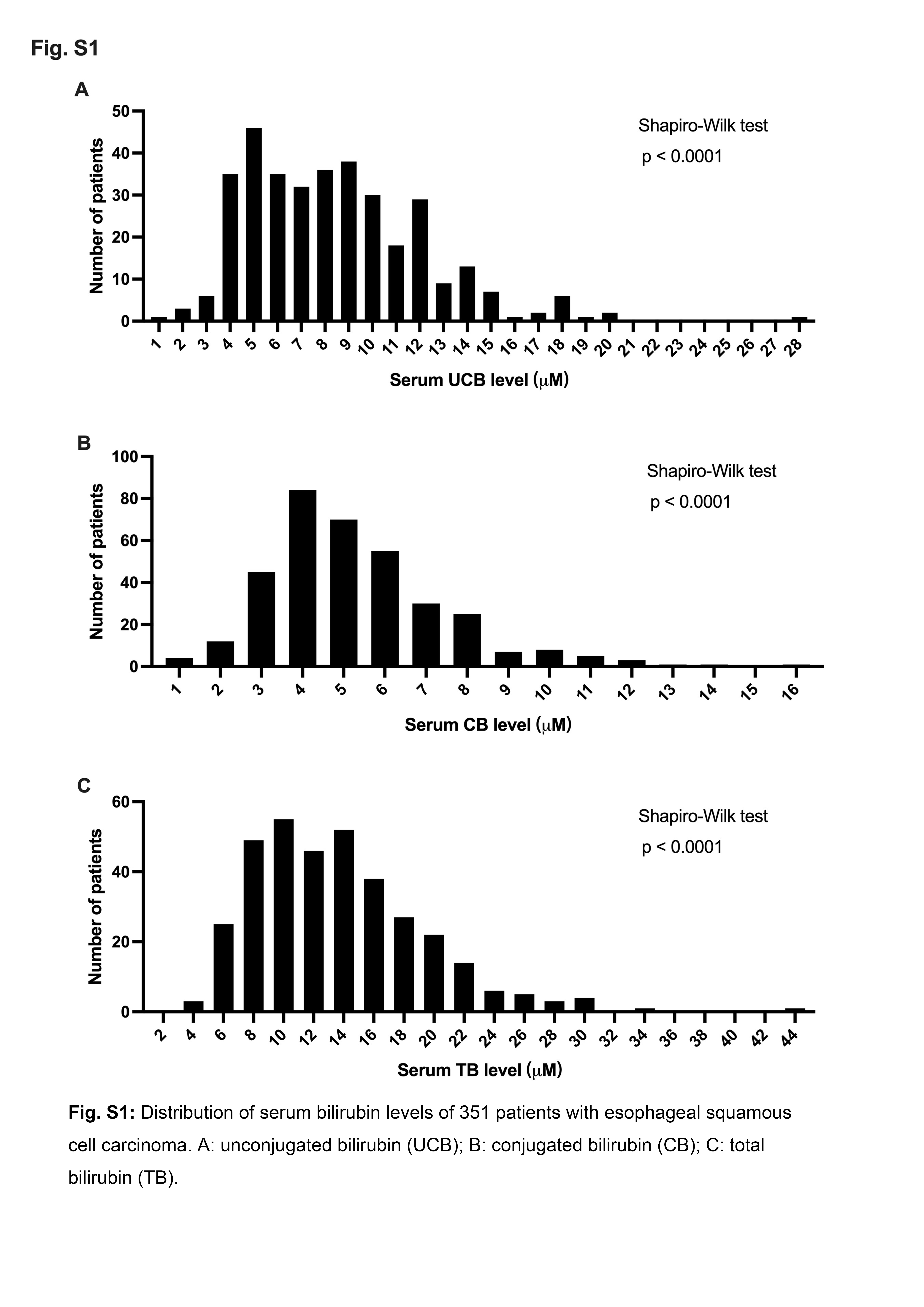

Supplement: Supplementary file 2 — Supplementary Material 2 [file 12885_2023_11215_MOESM2_ESM.tif]

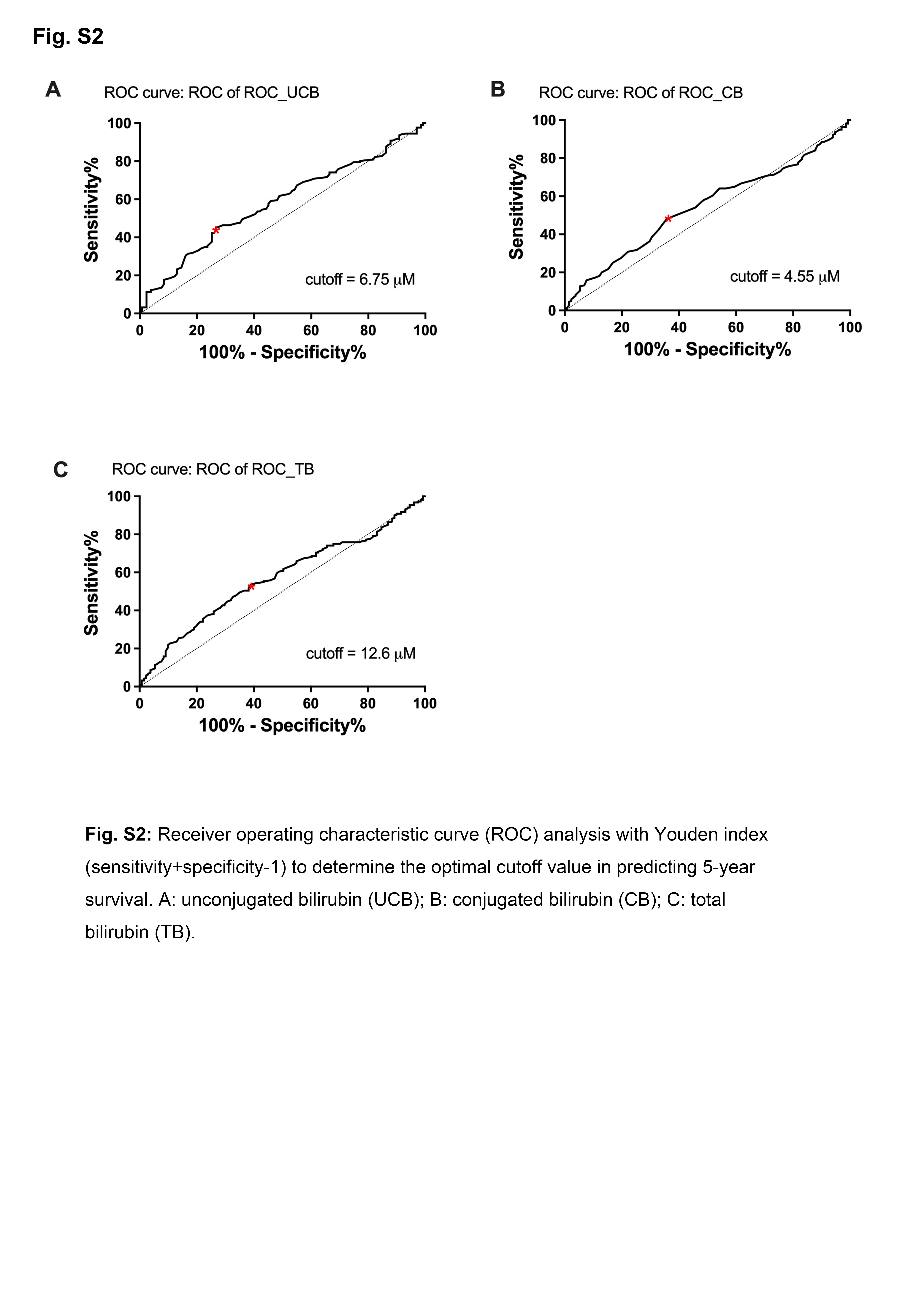

Supplement: Supplementary file 3 — Supplementary Material 3 [file 12885_2023_11215_MOESM3_ESM.tif]

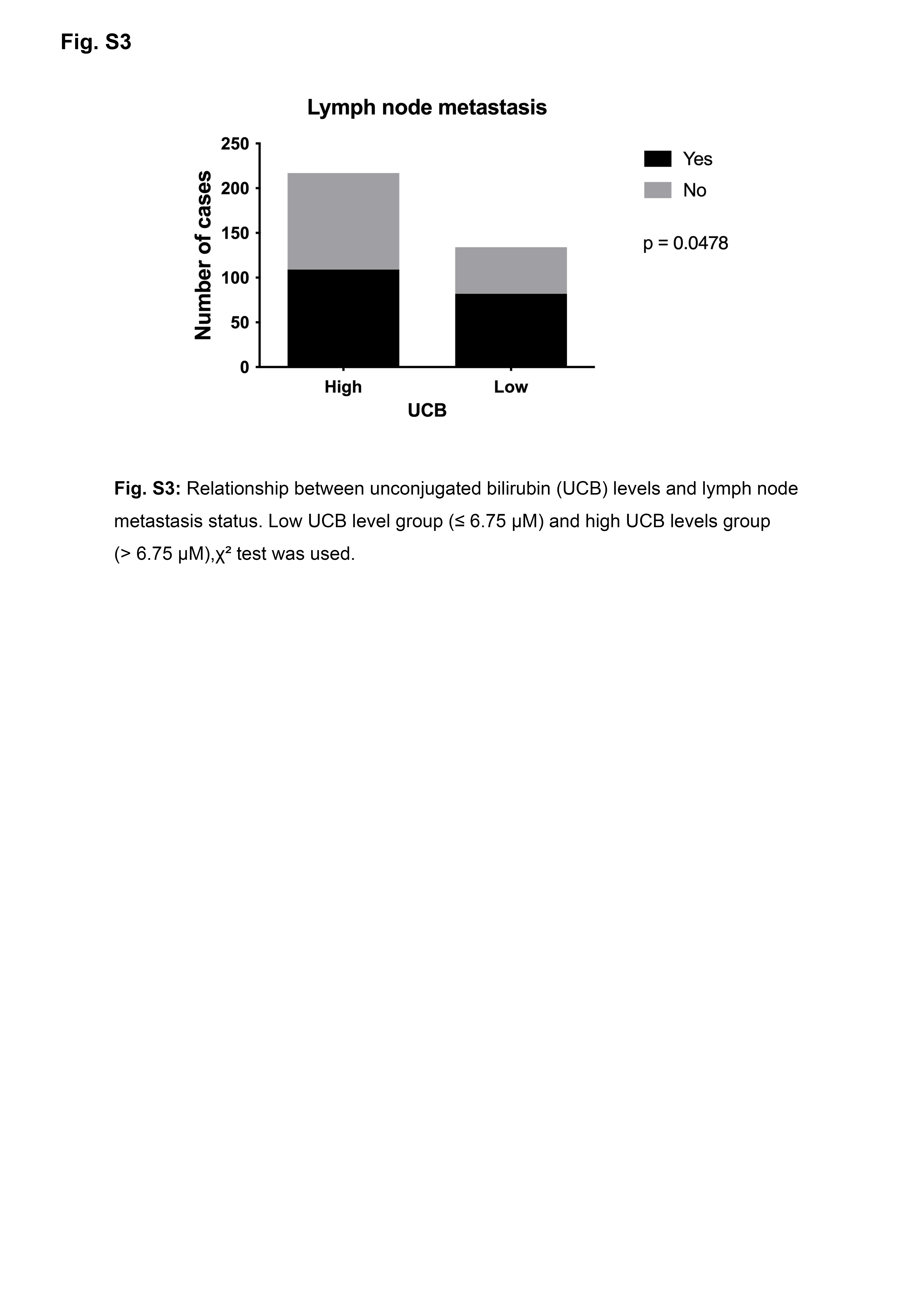

Supplement: Supplementary file 4 — Supplementary Material 4 [file 12885_2023_11215_MOESM4_ESM.tif]
